# Beyond the social gradient: the role of lifelong socioeconomic status in older adults’ health trajectories

**DOI:** 10.18632/aging.202342

**Published:** 2020-12-21

**Authors:** Lisa Harber-Aschan, Amaia Calderón-Larrañaga, Alexander Darin-Mattson, Xiaonan Hu, Laura Fratiglioni, Serhiy Dekhtyar

**Affiliations:** 1 Aging Research Center, Department of Neurobiology, Care Sciences and Society, Karolinska Institutet, Stockholm, Sweden; 2 Stockholm Gerontology Research Center, Stockholm, Sweden

**Keywords:** aging, health inequalities, latent class analysis, life-course, socioeconomic status

## Abstract

Inequalities in older adults’ health rarely consider life-course aspects of socioeconomic status (SES). We examined the association between lifelong SES and old-age health trajectories, and explored the role of lifestyle factors and depressive symptoms in this association. We followed 2760 adults aged 60+ from the Swedish National Study on Care and Aging, Kungsholmen. SES groups were derived using latent class analysis incorporating seven socioeconomic measures spanning childhood, midlife, and late life. We measured health using the Health Assessment Tool, which combines gait speed, cognition, multimorbidity, and disability. Linear mixed models were used to estimate health trajectories. Four SES groups were identified: High (34.9%), Middle (40.2%), Low (21.2%), and Mixed (3.8%). The Mixed group reported greater financial difficulties in childhood and older age, but varying SES attainment in midlife. Baseline health scores indicated that Mixed SES experienced substantial cognitive and physical deficits 12 years earlier than the High SES group. Compared to the High SES group, the Mixed SES group had the fastest health deterioration (β×time=−0.07, 95% CI:−0.11,−0.02); other groups followed a gradient (High>Middle>Low). Lifestyle factors and depressive symptoms attenuated the gradient but did not explain Mixed group’s health disadvantage. Life-long SES measures are crucial for understanding older adults’ health inequalities.

## INTRODUCTION

Socioeconomic inequalities in health are consistently observed and appear to persist into older ages [1, 2]. Socioeconomic status (SES) is a multifaceted concept incorporating multiple aspects of the social stratification process, including resource availability, power, and prestige [3]. Conventionally, SES has been captured using inter-related, yet conceptually distinct indicators, such as education, occupational class, or income, which influence health outcomes through different mechanistic pathways [4, 5]. However, other indicators of SES such as financial strain and wealth capture different dimensions of SES which may be especially relevant for older adults after retirement. Moreover, socioeconomic circumstances in childhood have been identified as important for health outcomes in old age [6], both through direct influences on late-life health, and indirect effects on downstream SES [7]. Nevertheless, much of the previous literature on health inequalities in older adults has not adequately considered the full complexity of SES, focusing only on a limited number of indicators and neglecting to consider the development of SES across multiple time-points throughout the life-course [7].

A life-course approach embraces the complexity of SES for understanding health in old age. It acknowledges that indicators are intertwined chronologically and that dynamic changes in SES may have important consequences for health in late life. Holistic assessments of SES throughout life are being increasingly used, with several recent studies highlighting the importance of childhood and adulthood SES indicators, and their interplay across life, for several health outcomes including disability, self-rated health, depression and cognition in old age [8–12]. Yet, even in life-course studies, SES measures from different life stages are often treated as distinct entities and their contribution to old-age health is estimated net of other life-course measures [11, 13]. However, given the inherent interrelatedness of socioeconomic indicators throughout life, investigating the joint effects of multiple SES indicators and their lifelong trajectories may be more informative than estimating the independent contribution of separate SES indicators.

In this study we use latent class analysis to identify people with similar socioeconomic profiles according to SES indicators spanning early life (childhood financial strain; parental occupational class), midlife (education, occupational class); and late life (homeownership, lack of financial assets, financial strain). Latent class analysis enables capturing the complexity of social position by simultaneously considering multiple socioeconomic indicators and identifying groups of individuals who share similar combinations of SES factors throughout life. To comprehensively capture health in old age, we investigate changes in the Health Assessment Tool (HAT) over 12 years, an integrated health measure in old age incorporating information not only on chronic disease, but also physical and cognitive function. Given the biological complexity and the heterogeneity of the ageing process, using multiple measures to reliably gauge health changes is crucial [14]. Our specific aims in this study were: 1) to identify distinct SES groups over the life-course; 2) to examine the association between the life-course SES groups and health changes over 12 years in individuals aged 60 years and older; and 3) to explore lifestyle factors and depressive symptoms as potential mechanisms in explaining any observed social gradient in older adults’ health.

## RESULTS

When deriving latent classes of lifelong socioeconomic position, the Akaike Information Criterion and Bayesian Information Criterion improved until the 4-class solution, and p-values for the Likelihood-Ratio test comparing the model to the saturated model increased substantially at 4-classes, indicating better model fit (Table 1). Given that the Bayesian Information Criterion did not improve further for five classes, and since the classes derived from the 4-class solution were more theoretically meaningful, we opted for four classes.

**Table 1:** Goodness-of-fit indices of latent class models with 2-5 classes.

	**AIC**	**BIC**	**X^2^ (*p*-value)**
2-class solution	24 753.52	24 877.90	897.74 (p<0.001)
3-class solution	24 483.91	24 673.45	606.13 (p<0.001)
4-class solution	24 293.95	24 548.64	394.17 (p=0.404)
5-class solution	24 268.82	24 588.66	347.04 (p=0.864)

Note: AIC, Akaike Information Criterion; BIC, Bayesian Information Criterion where smaller values indicate improved model fit; X^2^ statistic obtained from Likelihood-Ratio against saturated model, where p-values >0.05 indicate that the model fits as well as the saturated model.

Post-hoc posterior probabilities assigned individuals to four mutually exclusive SES groups labelled: High SES (n=795, 28.8%), Middle SES (n=1290, 46.7%), Low SES (n=572, 20.7%) and Mixed SES (n=103, 3.7%) (Table 2). The High SES group was characterized by professional parental occupations, postsecondary education (total: 97.2%; 6.6%, one or more years of higher education without university degree; 82.8%, undergraduate degree; 7.8%, postgraduate degree), professional occupations in mid-life, and limited financial difficulty in old age. The Middle SES group was characterized by parental non-manual occupations, high school education, non-manual midlife occupations, and limited financial difficulty in late life. Compared to High and Middle SES, twice as many in the Low SES group reported childhood financial strain. Most had parents with manual occupations, manual occupations themselves, and elementary education. The Mixed SES group was characterized by very high levels of financial strain, both in childhood and in older age, and had a substantially higher proportion of individuals reporting lack of financial assets and lack of homeownership compared to the other SES groups. However, their SES was mixed according to other indicators. In terms of parental occupational class and occupational class of the individual, the Mixed SES group slotted between the Middle and the Low SES group. Almost one-third of the Mixed group had postsecondary education – nearly double the proportion in the Middle SES group. Still a substantial proportion of the Mixed SES group also had elementary education (see Table 2 for detail).

**Table 2:** Distribution of socioeconomic indicators in the full sample and by the identified latent SES classes.

	**Full sample (N=2760)**		**High SES (n=795, 28.8%)**		**Middle SES (n=1290, 46.7%)**		**Low SES (n=572, 20.7%)**		**Mixed SES (n=103, 3.7%)**	
	**n**	**%**	**n**	**%**	**n**	**%**	**n**	**%**	**n**	**%**
Childhood financial strain										
No	2082	75.4	654	82.3	1042	80.8	338	59.1	48	46.6
Yes	678	24.6	141	17.7	248	19.2	234	40.9	55	53.4
Parental occupational class										
Manual	1056	38.3	96	12.1	496	38.5	411	71.8	53	51.5
Non-manual	1246	45.1	403	50.7	645	50.0	160	28.0	38	36.9
Professional	458	16.6	296	37.2	149	11.5	1	0.2	12	11.6
Education										
Elementary	390	14.1	3	0.4	0	-	369	64.5	18	17.5
High school	1365	49.5	19	2.4	1089	84.4	203	35.5	54	52.4
University (incl. incomplete)	1005	36.4	773	97.2	201	15.6	0	-	31	30.1
Occupational class										
Manual	564	20.4	0	-	133	10.3	387	67.7	44	42.7
Non-manual	1428	51.7	211	26.5	997	77.3	175	30.6	45	43.7
Professional	768	27.8	584	73.5	160	12.4	10	1.8	14	13.6
Lack of financial assets										
No	2576	93.3	790	99.4	1277	99.0	484	84.6	25	24.3
Yes	184	6.7	5	0.6	13	1.0	88	15.4	78	75.7
Financial strain										
No	2610	94.6	777	97.7	1253	97.1	571	99.8	9	8.7
Yes	150	5.4	18	2.3	37	2.9	1	0.2	94	91.3
Homeownership										
Rental/other	1364	49.4	198	24.9	691	53.6	383	67.0	92	89.3
Ownership	1396	50.6	597	75.1	599	46.4	189	33.0	11	10.7

Table 3 presents baseline characteristics of the study population according to SES groups. Notable differences across SES groups were observed with respect to sex, baseline age, civil status, country of birth, lifestyle factors, and depressive symptoms

**Table 3:** Baseline characteristics of the full sample and by latent SES groups.

	**Full sample (N=2760)**		**High SES (n=963, 34.9%)**		**Middle SES (n=1109, 40.2%)**		**Low SES (n=583, 21.2%)**		**Mixed SES (n=106, 3.8%)**		
	**n**	**%**	**n**	**%**	**N**	**%**	**n**	**%**	**n**	**%**	***p*^a^**
Sex											<0.001
Men	1048	38.0	408	51.3	410	31.8	197	34.4	33	32.0	
Women	1712	62.0	387	48.7	880	68.2	375	65.6	70	68.0	
Age cohort											<0.001
60	708	25.7	307	38.6	287	22.3	78	13.6	36	35.0	
66	538	19.5	177	22.3	268	20.8	74	12.9	19	18.5	
72	431	15.6	121	15.2	205	15.9	84	14.7	21	20.4	
78, 81	598	21.7	116	14.6	314	24.3	152	26.6	16	15.5	
84+	485	17.6	74	9.3	216	16.7	184	32.2	11	10.7	
Age, mean (SD)	72.3 (10.1)		68.7 (8.9)		72.7 (9.7)		77.1 (10.4)		69.7 (9.1)		<0.001
Civil status											<0.001
Married/partner	1302	47.2	507	63.9	588	45.6	196	34.3	11	10.7	
Unmarried	457	16.6	109	13.7	214	16.6	102	17.8	32	31.1	
Widowed/divorced	998	36.2	178	22.4	486	37.7	274	47.9	60	58.3	
Migrant											<0.001
No	2480	89.9	721	90.7	1174	91.0	501	87.6	84	81.6	
Yes	280	10.1	74	9.3	116	9.0	71	12.4	19	18.4	
Smoking											0.004
Never smoked	1250	45.5	330	41.6	587	45.7	292	51.3	41	40.2	
Smoked ever	1090	39.7	347	43.8	509	39.7	200	35.2	34	33.3	
Current smoker	407	14.8	116	14.6	187	14.6	77	13.5	27	26.5	
Alcohol use											<0.001
No or occasional	729	30.2	111	15.7	331	29.1	245	50.5	42	50.0	
Light or moderate	1277	52.9	438	61.8	611	53.7	199	41.0	29	34.5	
Heavy	409	16.9	160	22.6	195	17.2	41	8.5	13	15.5	
BMI, mean kg/m^2^ (SD)	25.7 (4.1)		25.6 (3.7)		25.8 (4.2)		25.8 (4.2)		26.9 (4.9)		0.016
Depressive symptoms, mean MADRS score^b^ (SD)	2.5 (3.7)		2.1 (3.1)		2.4 (3.6)		2.8 (3.9)		5.0 (6.1)		<0.001

^a^*p*-value for *X^2^* tests for categorical variables and ANOVA tests for continuous variables.

^b^Continuous MADRS score ranging from 0-60 where higher scores indicate greater and more severe depressive symptoms.

**Figure 1:**
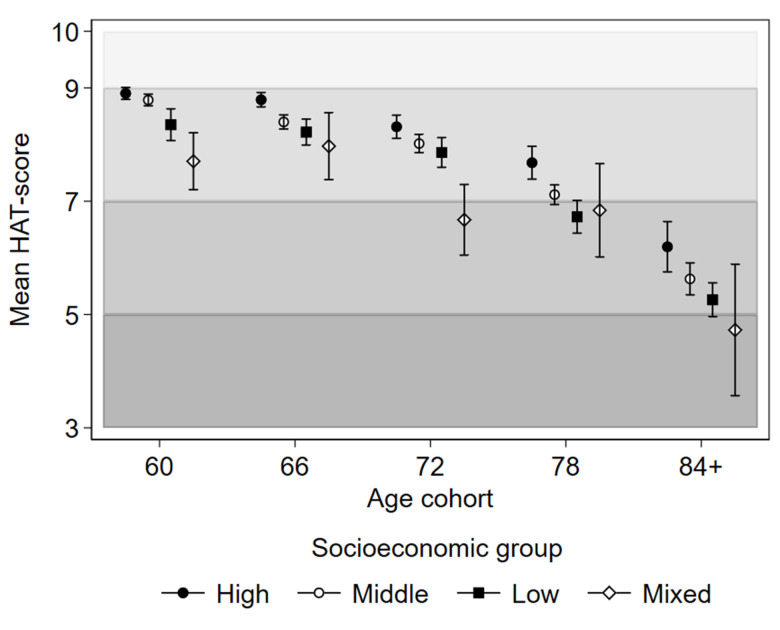
**Baseline health status as indicated by mean HAT-scores with 95% confidence intervals by latent socioeconomic groups and age cohorts of the Swedish National Study on Aging and Care in Kungsholmen, Stockholm, Sweden.** The shading of the graph represents the clinical characterization of HAT-scores: 3-4.9 mild functional dependence; 5-6.9 compromised physical functioning with multimorbidity with some cognitive deficits; 7-8.9 slight functional or cognitive impairments with some morbidities, and 9-10 good functioning and morbidity status.

Baseline health by SES group and age is illustrated in Figure 1. The average health status of Mixed SES individuals at age 60 was comparable to the health of High SES individuals at the age of 78. Substantial cognitive and physical deficits indicated by HAT scores <7 were on average experienced by the Mixed SES group already at 72 years, the Low SES group at 78 years, and the High SES group at 84 years.

**Figure 2:**
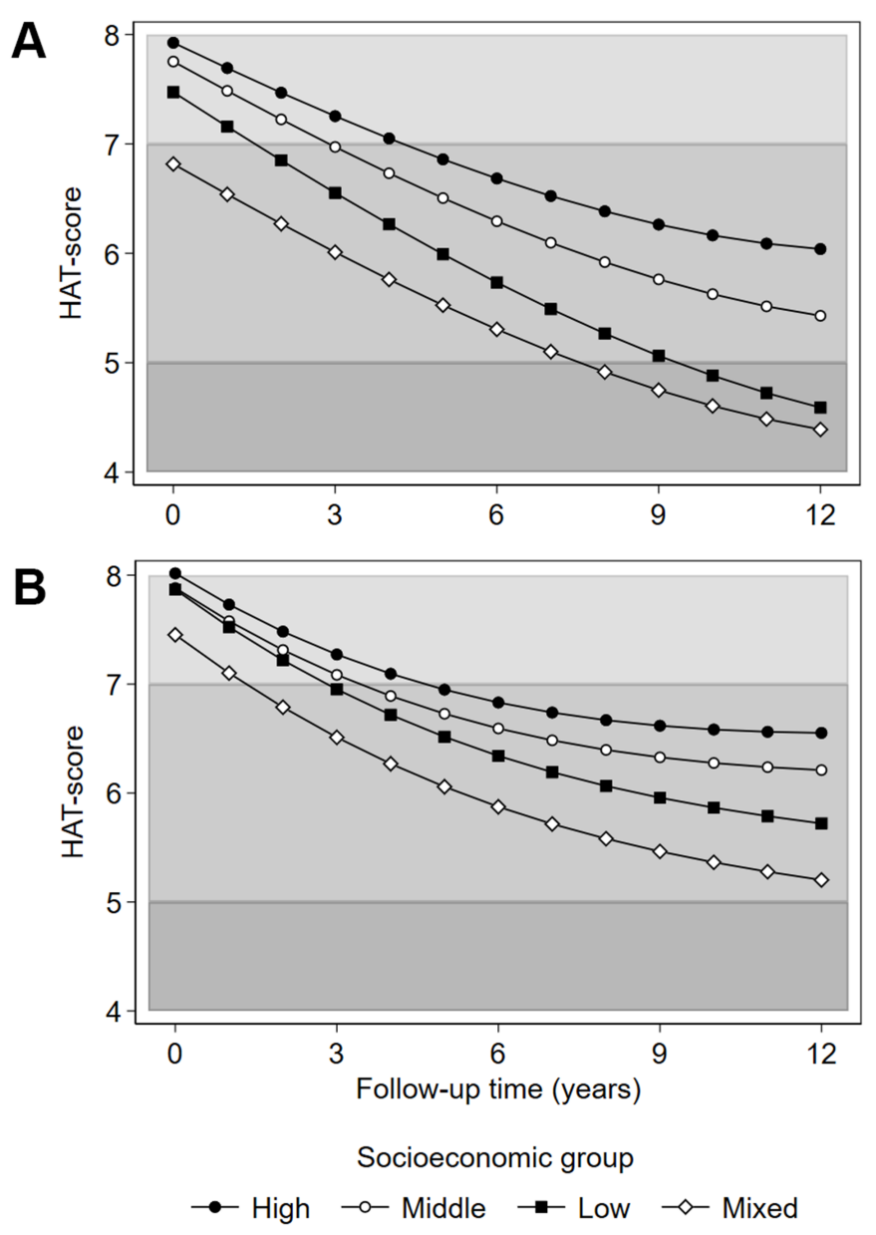
**Predicted HAT-score over 12 years of follow-up by latent socioeconomic groups.** (**A**) adjustment for age and sex; (**B**) adjustment for age, sex, civil status, migrant status, smoking, alcohol use, body mass index and depressive symptoms (Montgomery-Åsberg Depression Rating Scale score). Civil status, smoking, alcohol use, body mass index and depressive symptoms are time-varying covariates. The shading of the graph represents the clinical characterization of HAT-scores: 4-4.9 mild functional dependence; 5-6.9 compromised physical functioning with multimorbidity and some cognitive deficits; 7-8 slight functional or cognitive impairments with some morbidities.

Associations between SES groups and HAT over 12 years are presented in Table 4, and the predicted trajectories of health decline for SES groups are illustrated in Figure 2. In the age- and sex-adjusted model Mixed SES had the poorest health at baseline, followed by Low SES, relative to the High SES group, (Table 4, Model 1; Figure 2A). Low SES had the fastest health decline, and over time, the health trajectories of the Low and Mixed SES groups converged (Figure 2A), while the High and Middle SES groups had slower health deterioration.

**Table 4:** Associations between SES groups and health status at baseline (HAT-score) and HAT-score changes over 12 years.

	***β* (95% CI)**							
	**Model 1^a^**		**Model 2^b^**		**Model 3^c^**		**Model 4^d^**	
HAT score at baseline (ref: High SES)								
Middle SES	-0.17**	(-0.30 - -0.04)	-0.14**	(-0.27 - -0.01)	-0.11*	(-0.24 - 0.02)	-0.13**	(-0.26 - -0.01)
Low SES	-0.45***	(-0.62 - -0.29)	-0.39***	(-0.56 - -0.23)	-0.17*	(-0.33 - 0.00)	-0.15*	(-0.31 - 0.01)
Mixed SES	-1.11***	(-1.41 - -0.81)	-0.97***	(-1.27 - -0.66)	-0.71***	(-1.02 - -0.40)	-0.56***	(-0.87 - -0.26)
Slope of HAT score (ref: High SES)								
Middle SES * time	-0.04***	(-0.057 - -0.02)	-0.04***	(-0.06 - -0.02)	-0.03***	(-0.05 - -0.01)	-0.02**	(-0.03 - -0.001)
Low SES * time	-0.08***	(-0.110 - -0.06)	-0.08***	(-0.11 - -0.05)	-0.06***	(-0.09 - -0.04)	-0.06***	(-0.08 - -0.03)
Mixed SES * time	-0.05*	(-0.10 - 0.01)	-0.05*	(-0.10 - 0.01)	-0.06**	(-0.11 - -0.01)	-0.07***	(-0.11 - -0.02)
								
Observations		6,876		6,853		6,215		5,908
Number of individual respondents		2,716		2,715		2,493		2,467

^a^Adjusts for age and sex.

^b^Adjusts for age, sex, civil status, and migrant status.

^c^Adjusts for age, sex, civil status, and migrant status, smoking, alcohol use, and BMI

^d^Adjusts for age, sex, civil status, and migrant status, smoking, alcohol use, BMI, and depressive symptoms

Note: civil status, smoking, alcohol use, BMI and mental health were time-varying covariates; all models additionally adjust for quadratic and cubic time.

*** p<0.01, ** p<0.05, * p<0.1.

In models 2, 3 and 4 (Table 4) we performed stepwise adjustments to the association between SES groups and health. Civil status and migrant status only marginally affected the SES gradient. Lifestyle factors attenuated the health disadvantage of the Low, but not the Mixed SES group. Additional adjustment for depressive symptoms further attenuated the social gradient, although the health disadvantage of the Mixed SES group, both in terms of lowest baseline levels and fastest deterioration relative to the High SES group, remained (Table 4, Model 4; Figure 2B). Full model estimates are available in Supplementary Table 1.

We adjusted for time until study exit to examine whether selective attrition influenced observed associations. We also excluded those living in care homes, group living, or other institutional living arrangements (n=68; 2.5% of the sample). For both sets of sensitivity analyses, the principal results remained intact (analysis not shown).

We also attempted alternative clustering techniques using two unsupervised machine learning approaches: K-modes (with and without predefined starting vectors) and hierarchical clustering (divisive technique). A cluster representative of the Mixed SES group emerged in both K-modes with predefined starting vectors (selected based on the most prevalent SES characteristics in the four LCA groups), and in a hierarchical clustering approach. These additional analyses, however, proved sensitive to the selection of initializing points and introduced interpretational complexity by subdividing stable SES groups into theoretically unintuitive components. We opted for an LCA-based operationalization of SES groups, since it offers diagnostic statistics to identify the appropriate number of clusters, provides stronger interpretation ability, and easily incorporates variables at different scales.

Finally, we explored SES clustering with a data-dimension-reduction technique using multiple correspondence analysis. We also plotted the original data in a reduced two-dimensional grid (see Supplementary Figure 2). The results revealed that observations characterized by financial difficulty clustered in a distinctly different area of the 2-D grid compared to those defined by higher educational or occupational attainment. This analysis confirmed the importance of identifying a subset of the data defined by financial distress, which is also a defining characteristic of the Mixed SES group identified in LCA.

## DISCUSSION

In this longitudinal population-based study, we found four distinct SES groups: stable High, Middle and Low SES groups, and a Mixed SES group with varied education, occupational class, and parental occupational class, but distinctly characterized by financial difficulty in childhood and late life. Baseline health comparisons indicated that the Mixed SES group, on average, experienced substantial cognitive and physical deficits approximately 12 years earlier than the High SES group. Health trajectories over time followed an expected social gradient for the stable SES groups, while the Mixed SES group exhibited the fastest health decline. Lifestyle factors and depressive symptoms partially accounted for the health disadvantage of the Low SES group, while the Mixed SES group remained robust to adjustment for these factors. These results illustrate the importance of capturing socioeconomic heterogeneity across the life-course for understanding health inequalities in older age.

The three stable life-course SES groups were homogenous across all the socioeconomic indicators that we used, consistent with past research which has used similar methods to identify stable SES groups across the life-course [15]. We also identified a novel Mixed SES group which placed in between the Middle SES and Low SES groups on mid-life socioeconomic indicators but had the lowest SES according to all indicators captured in late life. This particular life-course SES trajectory has not yet been observed, but it is supported by previous research reporting that financial strain in childhood and across the life-course is important for health outcomes in old age [16, 17].

The observed health gradient across the stable High, Middle and Low SES groups is consistent with accumulating advantages and adversities over the life-course in relation to late-life health [6, 15, 18, 19]. The finding that health inequalities increased with age, contrasts some research reporting converging health trajectories between SES groups, a so-called leveling effect of age [2, 20]. Possible explanations may be that other studies relied on a limited number of conventional socioeconomic indicators such as education, occupational class and income that become increasingly irrelevant with age [4], used a unidimensional measure of health (e.g. morbidity), or had short follow-up durations. In contrast, our study used multiple socioeconomic indicators throughout life together with a health measure integrating cognitive and physical functioning, as well as morbidity, that was assessed over a prolonged period across a broad range of older ages.

The finding that the best health outcomes were observed in the High SES group, characterized by consistently high values across all life-course socioeconomic indicators, possibly suggests cumulative mechanisms of health advantage [18]. For example, high education is often a prerequisite for higher status occupations, providing a variety of health benefits, including health literacy, as well as avoidance of occupational health risks [3]. Furthermore, higher earnings from higher status occupations enable the accumulation of wealth, as evidenced by the large share of homeowners in the High SES group. Wealth, in turn, contributes to improved health not only through access to material resources, but also through avoidance of the psychosocial stress associated with socioeconomic deprivation [21].

The association between financial deprivation and poor health outcomes in older age has been reported previously [16, 17, 22, 23], especially in regions lacking universal health insurance [24]. It is, therefore, noteworthy that in the Swedish welfare context where state pensions and benefit systems ensure affordable medication and largely non-existent absolute poverty, the worst health outcomes were found in the Mixed SES group, predominantly characterized by financial strain. Already at baseline, the Mixed group had a mean score of HAT<7, indicating compromised physical functioning with multimorbidity and some cognitive deficits, while comparable health deficits occurred considerably later for the other SES groups. Given the steep rate of health deterioration, the Mixed SES group was also the first to cross the clinical HAT-threshold of 5, indicating functional dependence (a similar transition was only observed in the Low SES group, albeit one year later). Some have suggested that financial strain is especially detrimental to health in old age when it is persistent, as opposed to being experienced at critical windows [16], potentially reflecting the consequences of prolonged material and psychosocial deprivation [25]. Our results tentatively support this possibility, although we lacked an explicit measure of financial strain in midlife.

Lifestyle factors partially attenuated the health disadvantage of the stable Middle and Low SES groups (relative to High SES), which is consistent with the findings on the social patterning of behavioral risk factors [26]. Depressive symptoms also contributed to the health gradient across the stable SES groups, in line with prior findings that depression partially mediates health inequalities in older age [27]. In contrast, the health disadvantage of the Mixed SES group was robust to adjustment for covariates. One possibility could be stress and early adversity associated with childhood financial strain, which may have proximal consequences for health, as well as indirect influences on cascading social disadvantages in midlife [28]. Deprivation-driven psychosocial adversity and stress may further increase the likelihood of mental illness in adulthood [17, 29], whereas mental disorders are prospectively associated with both financial difficulty and poorer health in older age [30, 31]. While we adjusted for depressive symptoms in old age, depression earlier in life could nonetheless be a possibility. Finally, late-life social support and social network have been shown to influence health outcomes including multimorbidity, disability, and mortality in old age [32–35] and few in the Mixed SES group were married or had a partner. The interplay between financial loss, unemployment, or spousal loss could be a source of financial as well as psychosocial instability, especially considering the contrast between the Mixed and the Low SES group.

Our study has some limitations. The small size of the Mixed SES group combined with somewhat greater variability within the group, may have limited the precision of statistical comparisons. Notably, our results suggest that the difference between the Mixed and the High SES groups is substantial enough to be ascertained statistically, although there is a possibility that a less acute comparison between the Low and the Mixed group lacked power to be identified robustly. Importantly, our large overall sample enabled us to uncover this small, yet substantially disadvantaged group in terms of health outcomes. We encourage future studies with access to financial difficulty, both in childhood and late life, to verify our findings. The sample was obtained from a relatively affluent and homogenous area in Stockholm, Sweden. It is therefore likely that more heterogeneous classes would have been identified in a more diverse area, in line with research in urban areas [36]. Yet, even in this relatively homogenous sample we observed a clear social gradient, robust to adjustment of lifestyle factors and depressive symptoms. Self-reported measures of life-course SES may also be prone to recall bias, especially in elderly samples. We addressed this by excluding persons with diagnosed or probable dementia and cognitive impairment, but the possibility of misclassification remains. Furthermore, restricting the study population to cognitively intact participants without dementia, may have underestimated socioeconomic health inequalities, especially given the documented association between education and dementia [37]. Since we lacked information regarding midlife financial strain, this also limits any conclusions regarding the role of financial strain throughout the life-course. We also lacked information on health prior to old age and cannot rule out the possibility that poor health in childhood compromised socioeconomic attainment. One possibility could be to classify health status first and to search for matched SES characteristics within the HAT score strata, and other studies may consider exploring this approach.

## CONCLUSIONS

We identified stable life-course SES groups which followed an expected gradient in health, and an atypical SES trajectory characterized by financial strain which had the poorest health of all SES groups. This health disadvantage was not explained by lifestyle factors or depressive symptoms, potentially pointing towards the importance of psychosocial aspects of financial strain. Our study highlights the relevance of considering multiple socioeconomic indicators over the life-course to understand social inequalities in older adults’ health.

## MATERIALS AND METHODS

### Study design and population

We used data from the Swedish National study on Aging and Care, Kungsholmen, a population-based longitudinal study of adults aged 60 years or above living at home or in an institution in Kungsholmen, Stockholm, Sweden (N=3363) [38]. Participants were randomly sampled within 11 age-strata and assessed at baseline between 2001-2004 (participation rate: 73.3%) and at up to four follow-up points, equivalent to 12 years. We excluded those with dementia, intellectual disability, cognitive impairment (Mini-Mental State Exam scores <24) or with missing dementia information at baseline (n=380) to minimize recall bias. We subsequently excluded those with incomplete information on any of the SES indicators (n=223), producing an analytical sample of N=2760 (see Supplementary Figure 1). Ethical approval at each contact was obtained from the Regional Ethical Review Board in Stockholm. All study participants or their next of kin provided written informed consent.

### Measures

### Socioeconomic status (SES)

We used seven measures of life-course SES from the baseline nurse interview to derive the latent socioeconomic groups: financial strain and parental occupational class (childhood); education and occupational class (midlife); homeownership, lack of financial assets, and financial strain (late life). To measure childhood financial strain, participants were asked if their family struggled financially during their childhood (yes/no). The main occupations of participants’ parents until age 16 of participants were classified using the Swedish socioeconomic index of occupation, collapsing the derived categories into manual, non-manual, and professional, using the highest occupation of either parent. Education was categorized as elementary, secondary, or post-secondary/university. Participants’ longest held occupation was classified using the Swedish socioeconomic index and categorized into manual, non-manual, and professional. Lack of financial assets at the time of interview was captured by asking if respondents could pay an unexpected expense of 14,000 SEK (approximately €1330) within a week. Financial strain was assessed by inquiring about difficulties to keep up with payments (e.g. rent or bills) in the past 12 months. Homeownership distinguished between those owning their property versus rentals or other forms of housing.

### Health

The Health Assessment Tool (HAT) provided a comprehensive assessment of older adults’ health [39, 40]. HAT combines five domains of health and functioning: 1) limitations in activities of daily living (ADL) (e.g. bathing, dressing), 2) limitations in instrumental ADL (e.g. grocery shopping, housekeeping), 3) cognitive functioning as assessed by the Mini-Mental State Exam 4) gait speed as assessed by time to walk 6 meters or 2.44 meters if walking difficulty was reported, and 5) chronic multimorbidity as a count of conditions that are prolonged and either leave residual disability, adversely affect quality of life, or require substantial care (918 chronic disease codes from the International Statistical Classification of Diseases and Related Health Problems 10^th^ Revision, belonging to 60 disease categories identified from clinical assessments, laboratory tests, drug use, and patient records) [41]. HAT-scores were computed using nominal response models, producing an overall index score of 0-10 with clinical cut-offs of: 0-1.9: severe functional dependence; 2-4.9: mild functional dependence; 5-6.9: compromised physical functioning with multimorbidity and some cognitive deficits; 7-8.9 slight functional or cognitive impairments with some morbidities; and 9-10: good functioning and morbidity status [39].

### Covariates

Demographic covariates included sex, age, civil status (married/cohabiting with partner, widowed, unmarried, and divorced), and immigrant status (Swedish born vs. foreign-born). Smoking was categorized as: never smoked, smoked in the past, and current smokers. Alcohol use was coded as no/occasional, light/moderate, and heavy, taking both frequency and quantity of consumption into consideration, computed separately for men and women. BMI was derived from height and weight measures (kg/m^2^). Depressive symptoms were assessed using the Montgomery-Åsberg Depression Rating Scale; a subscale of the Comprehensive Psychopathological Rating Scale validated for older adults [42, 43]. Civil status, smoking, alcohol use, BMI, and depressive symptoms were assessed at baseline and each follow-up point and were time-varying covariates.

### Analysis

### Estimating latent socioeconomic groups

Latent class analysis, a statistical person-centered approach which groups individuals into unobserved classes based on responses to manifest variables [44], was used to derive the latent SES groups from the seven socioeconomic indicators. The Stata 15 command *gsem* *lclass* estimated the latent class models using maximum-likelihood estimation [45]. We began with a two class-solution, incrementally increasing the number of classes. Statistical criteria and interpretations based on subject matter knowledge determined the final choice of latent classes. Goodness-of-fit indices included Akaike Information Criterion [46], Bayesian Information Criterion [47], and the Likelihood-Ratio Test. Participants were assigned to one class according to their highest posterior probability [48]. Derived classes were described according to the socioeconomic indicators contributing to the latent class analysis, and other baseline characteristics.

### Associations between latent socioeconomic groups and health

Linear mixed models estimated trajectories of health change for the derived latent socioeconomic groups. Interactions between follow-up time and socioeconomic groups were included as fixed effects, and random effects for individual and follow-up time were included, using an unstructured covariance structure and a restricted maximum likelihood estimation method, and including a quadratic and a cubic term for time, allowing for accelerated health change curves. Models incrementally adjusted for demographic indicators, lifestyle factors, and depressive symptoms.

## Supplementary Material

Supplementary Figures

Supplementary Table 1
