# Supplementary material for: Beyond the social gradient: the role of lifelong socioeconomic status in older adults’ health trajectories
Source: Aging (Albany NY). 2020 Dec 21;12(24):24693–708. doi: 10.18632/aging.202342 (PMC7803509; doi:10.18632/aging.202342)
Supplement: Supplementary Figures [file aging-12-202342-s001.pdf]

## SUPPLEMENTARY FIGURES

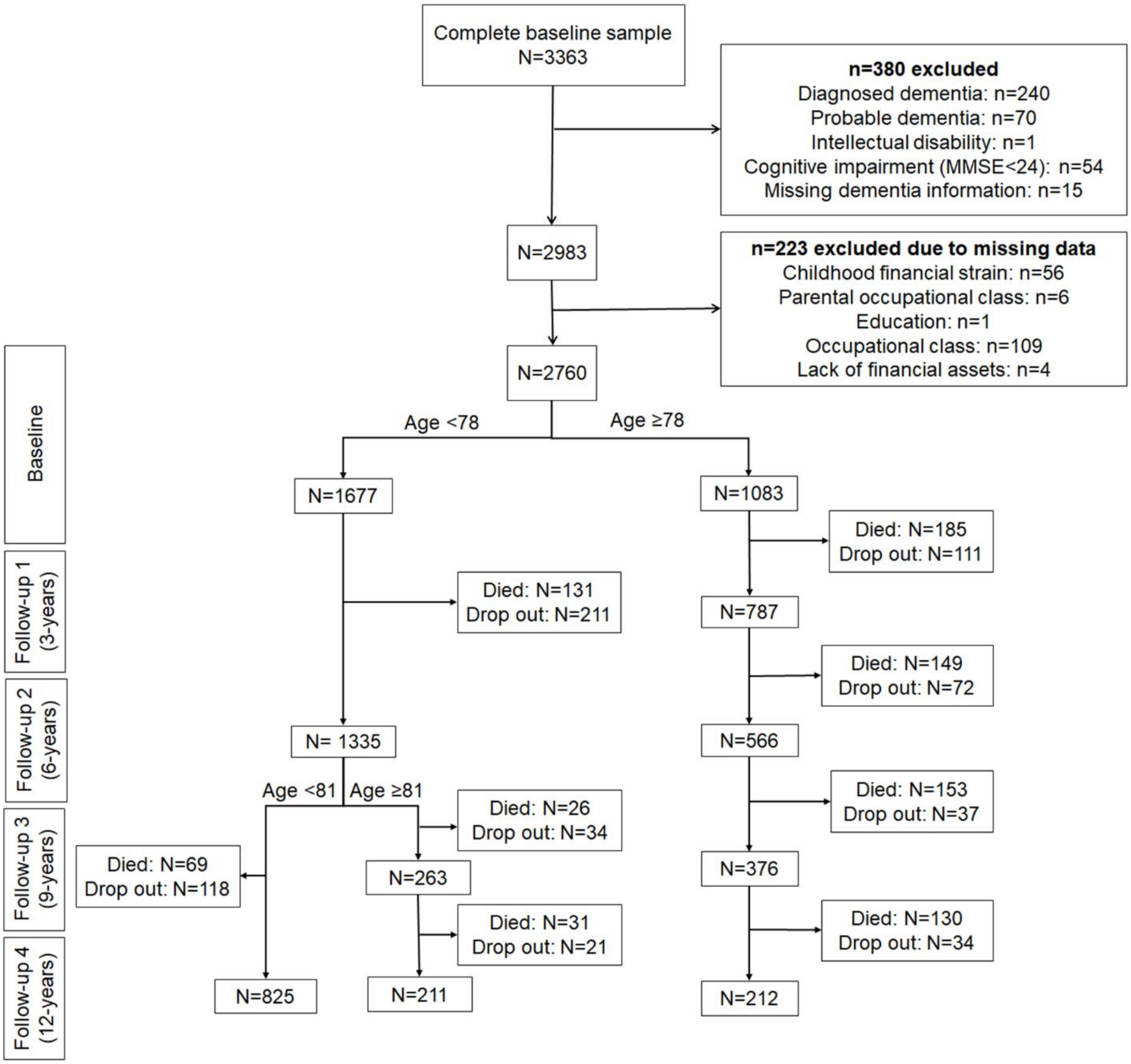


**Supplementary Figure 1. Process of deriving the analytical sample from the complete baseline sample and attrition at each follow-up point for the Swedish National Study on Aging and Care in Kungsholmen, Stockholm, Sweden (2001-2013).**

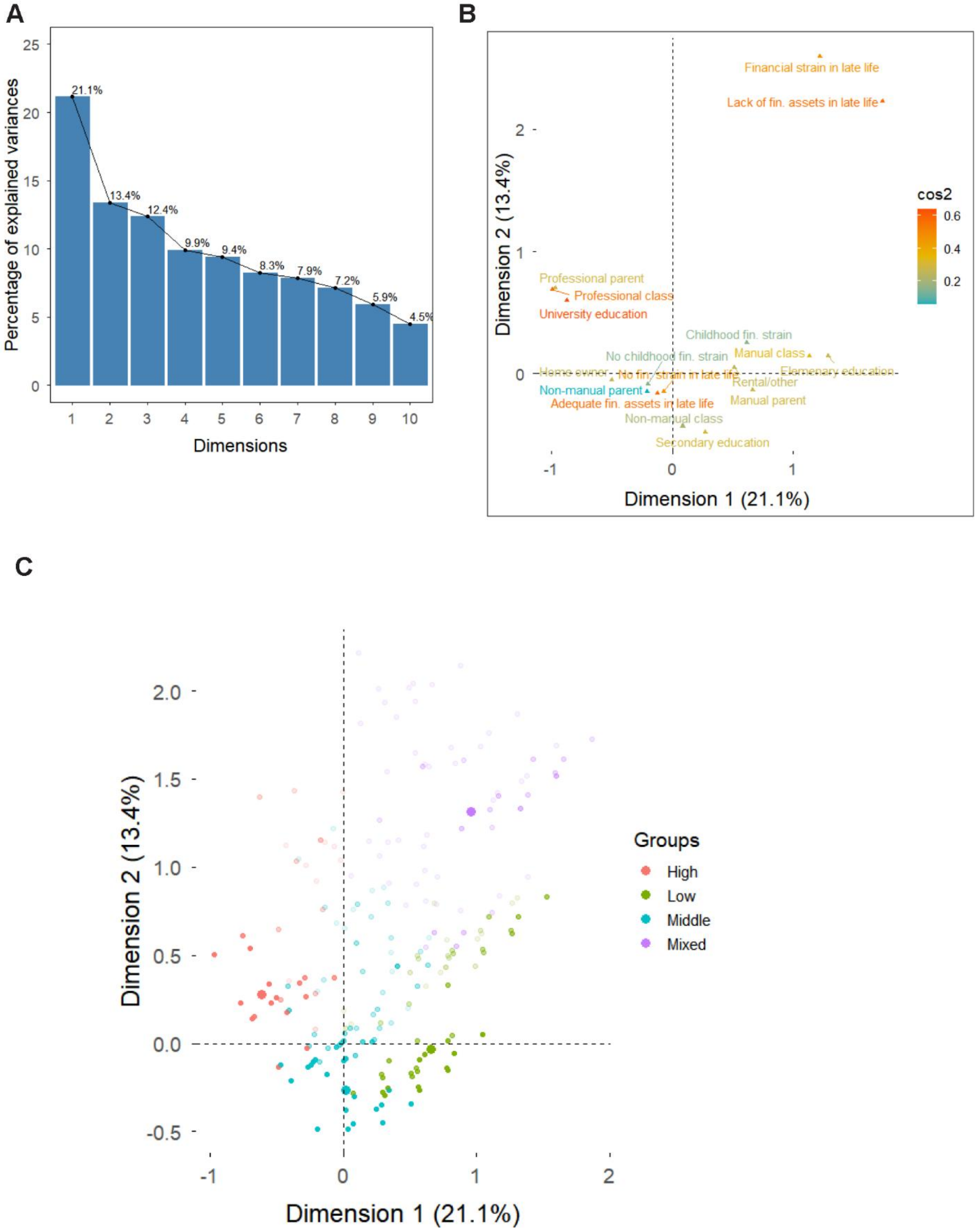


**Supplementary Figure 2. Data dimension reduction and 2-D plots of SES groups.** (**A**) Variance retained by different data dimensions following multiple correspondence analysis. (**B**) The coordinates of SES categories on 2-D grid after reduction. Color gradient indicates the contribution of categories to specific data dimensions. (**C**) The coordinates of observations after data dimension reduction. Highlight gradient is assigned in accordance with the SES group (based on LCA) corresponding to that observation. Thickness of the dots is proportional to the number of observations located on a given plot of 2-D space.
