# Supplementary material for: Beyond the social gradient: the role of lifelong socioeconomic status in older adults’ health trajectories
Source: Aging (Albany NY). 2020 Dec 21;12(24):24693–708. doi: 10.18632/aging.202342 (PMC7803509; doi:10.18632/aging.202342)
Supplement: Supplementary Table 1 [file aging-12-202342-s002.pdf]

SUPPLEMENTARY TABLE

Supplementary Table 1. Complete estimates of covariates in models testing associations between socioeconomic groups and speed of health change (HAT-score) over 12 Years.

	β (95% CI)							
	**Model 1[a,e]**		**Model 2[b,e]**		**Model 3[c,e]**		**Model 4[d,e]**	
HAT-score at baseline								
High SES	0	Referent	0	Referent	0	Referent	0	Referent
Middle SES	-0.17[h]	(-0.30, -0.04)	-0.14[h]	(-0.27, -0.01)	-0.11[g]	(-0.24, 0.02)	-0.13[h]	(-0.26, -0.01)
Low SES	-0.45[i]	(-0.62, -0.29)	-0.39[i]	(-0.56, -0.23)	-0.17[g]	(-0.33, 0.00)	-0.15[g]	(-0.31, 0.01)
Mixed SES	-1.11[i]	(-1.41, -0.81)	-0.97[i]	(-1.27, -0.66)	-0.71[i]	(-1.02, -0.40)	-0.56[i]	(-0.87, -0.26)
Slope of HAT-score								
High SES	0	Referent	0	Referent	0	Referent	0	Referent
Middle SES * time	-0.04[i]	(-0.06, -0.02)	-0.04[i]	(-0.06, -0.02)	-0.03[i]	(-0.05, -0.01)	-0.02[h]	(-0.03, -0.00)
Low SES * time	-0.08[i]	(-0.11, -0.06)	-0.08[i]	(-0.11, -0.05)	-0.06[i]	(-0.09, -0.04)	-0.06[i]	(-0.08, -0.03)
Mixed SES * time	-0.05[g]	(-0.10, 0.01)	-0.05[g]	(-0.10, 0.01)	-0.06[h]	(-0.11, -0.01)	-0.07[i]	(-0.11, -0.02)
Time	-0.24[i]	(-0.30, -0.17)	-0.23[i]	(-0.30, -0.16)	-0.26[i]	(-0.33, -0.19)	-0.31[i]	(-0.38, -0.24)
Sex								
Male	0	Referent	0	Referent	0	Referent	0	Referent
Female	-0.19[i]	(-0.30, -0.09)	-0.13[h]	(-0.24, -0.02)	-0.10[g]	(-0.21, 0.01)	-0.08	(-0.19, 0.02)
Age (years)	-0.12[i]	(-0.13, -0.12)	-0.12[i]	(-0.13, -0.12)	-0.11[i]	(-0.12, -0.11)	-0.11[i]	(-0.12, -0.10)
Civil status								
Married			0	Referent	0	Referent	0	Referent
Unmarried			-0.43[i]	(-0.57, -0.28)	-0.30[i]	(-0.44, -0.15)	-0.24[i]	(-0.38, -0.11)
Widowed/Divorced			-0.20[i]	(-0.31, -0.10)	-0.12[h]	(-0.22, -0.01)	-0.10[h]	(-0.20, -0.001)
Migrant (vs. non-migrant)			-0.16[g]	(-0.33, 0.01)	-0.08	(-0.24, 0.09)	-0.04	(-0.20, 0.12)
Smoking								
Never smoked					0	Referent	0	Referent
Smoked ever					-0.17[i]	(-0.28, -0.06)	-0.14[i]	(-0.24, -0.04)
Current smoker					-0.22[i]	(-0.37, -0.07)	-0.19[i]	(-0.33, -0.05)
Alcohol use								
No or occasional					0	Referent	0	Referent
Light or moderate					0.54[i]	(0.45, 0.64)	0.41[i]	(0.32, 0.51)

Heavy					0.60[i]	(0.47, 0.73)	0.48[i]	(0.36, 0.60)
BMI (kg/m^2)					0.007	(-0.005, 0.02)	-0.02[i]	(-0.03, -0.01)
Depressive symptoms[f]							-0.08[i]	(-0.09, -0.07)
Observations	6,876		6,853		6,215		5,908	
Number of individual respondents	2,716		2,715		2,493		2,467	

Abbreviations: BMI, Body Mass Index; CI, confidence interval; HAT, Health Assessment Tool; SES, socioeconomic status

[a]Adjusts for age and sex.

[b]Adjusts for age, sex, civil status (time-varying covariate), and migrant status.

[c]Adjusts for age, sex, civil status, migrant status, smoking, alcohol use, and BMI (civil status, smoking, alcohol use and BMI are time-varying covariates)

[d]Adjusts for age, sex, civil status, migrant status, smoking, alcohol use, and BMI, and depressive symptoms (civil status, smoking, alcohol use, BMI and depressive symptoms are time-varying covariates)

[e]All models additionally adjust for quadratic and cubic time.

[f]Continuous score from the Montgomery-Åsberg Depression Rating Scale ranging from 0-60 where higher scores indicate greater and more severe depressive symptoms.

[g]*P*<0.1.

[h]*P* <0.05.

[i]*P* <0.01.
